# A Case of Lung Abscess Caused by Double Immunosuppressive Therapy to Treat Ulcerative Colitis

**DOI:** 10.3390/medicina56110595

**Published:** 2020-11-07

**Authors:** Keiichi Tominaga, Mimari Kanazawa, Takanao Tanaka, Shunsuke Kojimahara, Takeshi Sugaya, Shoko Watanabe, Akira Yamamiya, Yuichi Majima, Makoto Iijima, Kenichi Goda, Atsushi Irisawa

**Affiliations:** Department of Gastroenterology, Dokkyo Medical University, 880 Kitakobayashi, Mibu, Tochigi 321-0293, Japan; mimari77@dokkyomed.ac.jp (M.K.); tana1986@dokkyomed.ac.jp (T.T.); s-koji@dokkyomed.ac.jp (S.K.); t-sugaya@dokkyomed.ac.jp (T.S.); shoko-t@dokkyomed.ac.jp (S.W.); akira-y@dokkyomed.ac.jp (A.Y.); y-majima@dokkyomed.ac.jp (Y.M.); mkiijima@dokkyomed.ac.jp (M.I.); goda@dokkyomed.ac.jp (K.G.); irisawa@dokkyomed.ac.jp (A.I.)

**Keywords:** ulcerative colitis, lung abscess, Janus kinase inhibitor, prednisolone

## Abstract

A 25-year-old man was admitted to our institution for remission induction therapy to treat a 12-year condition of ulcerative colitis (UC). Previously, he was treated with drugs, such as mesalamine, immunomodulators, prednisolone (PSL), and anti-TNFα anti-body, but remission was not maintained. Therefore, we started remission induction therapy with 20 mg/day of tofacitinib (TOF) to inhibit the action of Janus kinase. On the 29th day after TOF administration, he developed a lung abscess with high fever. A chronic bulla was already present in his lung; therefore, the lung abscess was likely formed due to a combination of the bulla being present and the pharmacological effects of TOF. Our report is significant as it highlights the compounding association between TOF and PSL therapy and bulla presence with the rare adverse effect of developing an abscess.

## 1. Introduction

Ulcerative colitis (UC) is a chronic inflammatory bowel disease of unknown etiology [1,2]. In recent years, many studies have demonstrated that treatments targeting inflammatory cytokines are effective for UC [3,4]. In patients undergoing refractory biological treatment, combinations of two or three immunomodulatory drugs, such as prednisolone (PSL), immunomodulators, and anti-tumor necrosis factor (TNF)-α inhibitors, are used. Although serious infections might be induced due to immunosuppressive therapy, the occurrence of lung abscess as an adverse event is rare. We reported a case of lung abscess in a patient with UC with a pulmonary bulla, who was undergoing double immunosuppressive therapy with tofacitinib (TOF) and PSL to facilitate remission of UC.

## 2. Presentation of Case Report

A 25-year-old man was admitted to our hospital for the treatment of a 12-year condition of UC. He was a non-smoker and had no syndrome. Previously, he was treated with drugs, such as mesalamine, immunomodulators, prednisolone, and the anti-TNFα anti-body, but remission was not maintained. He had been undergoing treatment with 5–10 mg/day of PSL for 10 years since the onset of UC. At our hospital, he was administrated TOF (20 mg/day) in order to withdraw PSL and eliminate PSL-dependent UC. However, even after remission therapy with TOF, his symptoms did not improve. On the 9th day of treatment, the abdominal symptoms worsened, and he was hospitalized. Table 1 shows the patient’s laboratory data at admission.

**Figure 1:**
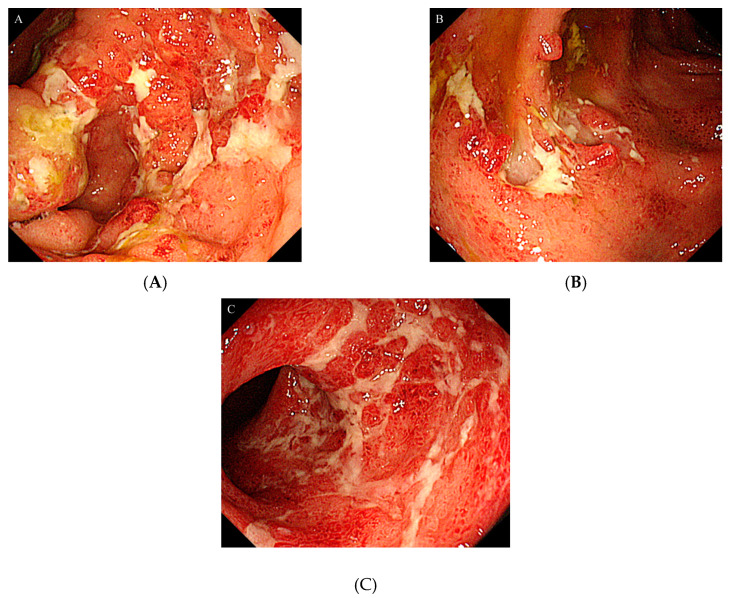
Colonoscopy showed severe inflammation in the colon mucosa: (**A**) Ascending colon; (**B**) Transverse colon; (**C**) Sigmoid colon.

After admission, a colonoscopy was performed, which revealed severe inflammation in the colon with marked erythema, absent vascular pattern, friability, erosions, and deep punched-out ulcers present along the cecum to the descending colon (Figure 1 A–C). The cytomegalovirus (CMV) antigenemia was negative; however, based on endoscopic findings and PSL dependence, he was diagnosed with infectious colitis and active UC due to reactivation of CMV. Therefore, ganciclovir was administrated immediately. The patient’s long-term use of PSL was considered to be the primary cause of CMV colitis.

**Figure 2:**
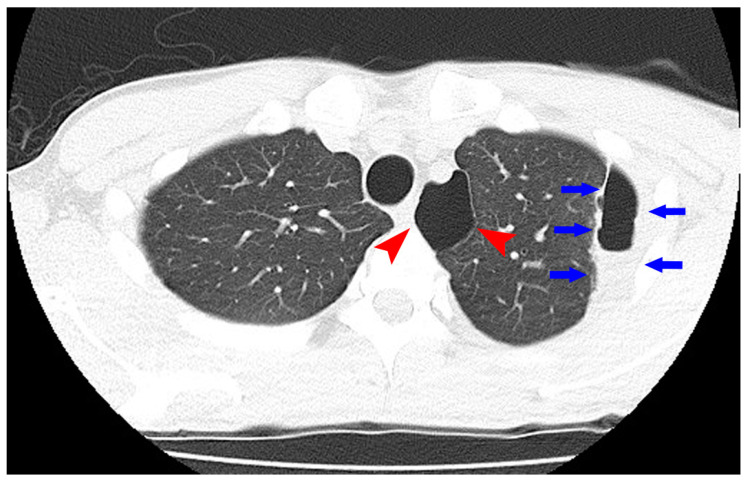
Computed tomography of the chest showed an emphysematous bulla (Arrowhead) in the upper left lobe of the lung and fluid formation inside the bulla (Arrow).

**Figure 3:**
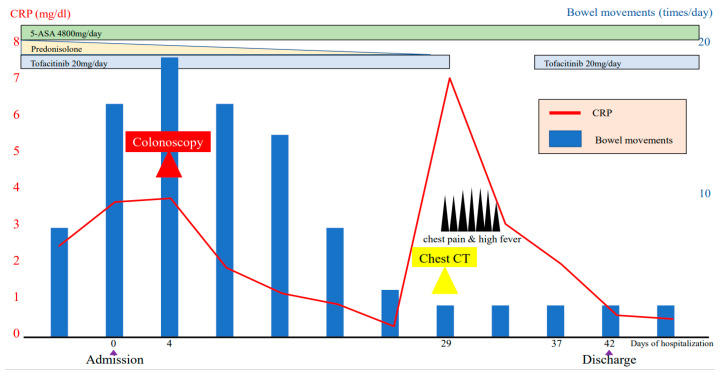
Clinical course of the patient’s condition.

**Figure 4:**
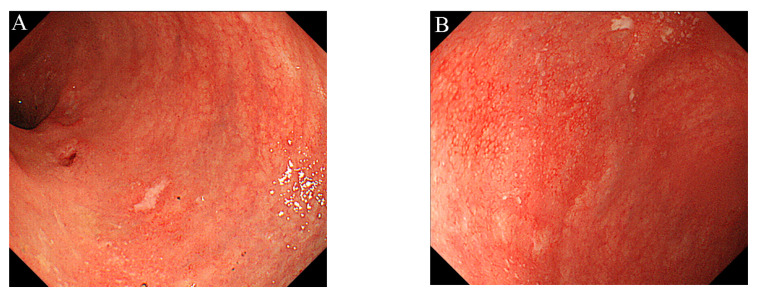
Colonoscopy showed mild inflammation in the colorectal mucosa, after one year of treatment with tofacitinib alone: (**A**) Sigmoid colon; (**B**) Rectum.

To maintain the remission, 20 mg/day of TOF was continued. His general condition improved, and discharge was considered. Suddenly, on the 29th day of hospitalization, he developed fever and chest pain. Serum C-reactive protein level was markedly increased. Suddenly, he developed fever and chest pain on Day 29. Serum C-reactive protein level was markedly increased. Computed tomography (CT) of the chest showed an emphysematous bulla in the upper left lobe of the lung and fluid formation inside the bulla (Figure 2). He was diagnosed with lung abscess by a pulmonologist and was started on meropenem. Table 2 shows the patient’s laboratory data at the onset of lung abscess. TOF was discontinued. The treatment was successful, and the imaging findings improved. He resumed TOF on Day 37 of his hospital stay and was discharged on Day 42. At present, his remission is maintained with continued with TOF alone, and he has not experienced any adverse event, including a relapse of the lung abscess. The patient’s clinical course is shown in Figure 3. One year after being treated with TOF alone, the endoscopic findings of mucosal inflammation had improved (Figure 4 A,B).

## 3. Discussion

In a patient with UC, induction and maintenance of remission are fundamental to successful treatment, and immunosuppressive therapy is often required to achieve this. PSL is a frequently used for remission induction therapy of UC and is effective in about 80% of cases [5]. Thus, PSL is recognized as the first-line treatment drug for 5-aminosalicylic acid (5-ASA)-resistant UC. Anti-TNF-α antibody, vedolizumab, tacrolimus or TOF may be used when if remission induction therapy with PSL fails [6]. TOF is the molecule inhibitor of Janus kinase (JAK) and is a potent drug that is used to suppress enteritis by blocking the intracytoplasmic signaling of many cytokines involved in the pathogenesis of UC [7]. TOF is especially expected to have an effect on UC cases in which the anti-TNFα antibody was unresponsive [8].

The effectiveness of TOF for UC has been reported in a worldwide multi-center study: the OCATVE study [9]. The safety of TOF was also evaluated in this study, and no significant difference was observed between the placebo group and TOF group for serious adverse events in any of the safety evaluations conducted via remission induction and maintenance trials. However, the onset of infections, such as *Clostridioides difficile* infection, pneumonia, cellulitis, and anal abscess, tended to be higher in the TOF group than in the placebo group during the remission induction (18.2–23.2%) and maintenance (35.9 to 39.8%) trials. This indicated that TOF-associated infections need to be carefully considered.

Our patient developed a lung abscess during the administration of both PSL and TOF. Various adverse events associated with PSL administration, such as moon-face, hyperglycemia, infection, and osteoporosis, have been observed frequently [10]. Moreover, although it is recognized that the adverse effects of TOF are generally mild in previous studies [11], several viral and bacterial infections have also been reported [8,12,13]. In our patient, we believed that the development of lung abscess was related to the use of two immunosuppressants.

Generally, while discontinuing PSL administration in a patient with refractory PSL, it is necessary to withdraw PSL gradually. Therefore, the next immunosuppressive agent should be administered before PSL is withdrawn, resulting in a period of time where two drugs are used together. In our patient, a lung abscess developed in the duration where both PSL and TOF were being consumed. However, after PSL withdrawal and during remission maintenance treatment with only TOF, no serious infection, was observed. Thus, in our case, it was considered that the lung abscess was caused by excessive immunosuppression due to the combination of PSL and TOF. In addition, our patient had an emphysematous bulla that presented a risk for lung abscess. Therefore, the presence of the bulla might have propounded the development of the lung abscess under excessive immunosuppression.

## 4. Conclusions

Our report highlights the importance of evaluating the risk of various infections by confirming comorbidities and conducting screening tests such via imaging modalities (e.g., chest X-ray, CT, etc.) before starting excessive immunotherapy.

## Figures and Tables

**Table 1:** Laboratory data on admission.

AST	15 U/L	WBC	12.4 × 10^9^/L
ALT	11 U/L	RBC	3.92 × 10^12^/L
ALP	158 U/L	Hb	12.2 g/dL
γGTP	15 U/L	Plt	50.6 × 10^4^/L
T-Bil	0.4 mg/dL	ESR (1 h)	45 mm
UN	10 mg/dL		
Cre	0.67 mg/dL	T-SPOT^®^	(-)
TP	7.3 mg/dL	HBsAg	(-)
Alb	3.5 mg/dL	HBcAb	(-)
CRP	3.8 mg/dL	CMV antigenemia	(-)
Stool culture	Normal		

Abbreviations: AST: Aspartate aminotransferase; ALT: Alanine aminotransferase; ALP; Alkaline phosphatase; γGTP: γ-glutamyl transpeptidase; T-Bil: Total bilirubin; UN: Urea nitrogen; Cre: Creatinine; TP: Total protein; Alb: Albumin; CRP: C-reactive protein; WBC: White blood cell; RBC: Red blood cell; Hb: Hemoglobin; Plt: Platelet; ESR (1 h): Erythrocyte sedimentation rate (1 h); HBsAg: Hepatitis B surface antigen; HBcAb: Hepatitis B core antibody; CMV: Cytomegalovirus.

**Table 2:** Laboratory data at the onset of lung abscess.

AST	15 U/L	WBC	11.3 × 10^9^/L
ALT	19 U/L	Neutro	72.2%
ALP	164 U/L	Lympho	19.0%
γGTP	19 U/L	RBC	3.49 × 10^12^/L
T-Bil	0.7 mg/dL	Hb	11.1 g/dL
UN	7 mg/dL	Plt	39.9 × 10^4^/L
Cre	0.68 mg/dL	ESR (1 h)	58 mm
TP	7.6 mg/dL		
Alb	3.5 mg/dL		
CRP	6.98 mg/dL		
